# A Novel Histogram Region Merging Based Multithreshold Segmentation Algorithm for MR Brain Images

**DOI:** 10.1155/2017/9759414

**Published:** 2017-03-16

**Authors:** Siyan Liu, Xuanjing Shen, Yuncong Feng, Haipeng Chen

**Affiliations:** ^1^Key Laboratory of Symbolic Computation and Knowledge Engineering of the Ministry of Education, Jilin University, Changchun 130012, China; ^2^College of Computer Science and Technology, Jilin University, Changchun 130012, China

## Abstract

Multithreshold segmentation algorithm is time-consuming, and the time complexity will increase exponentially with the increase of thresholds. In order to reduce the time complexity, a novel multithreshold segmentation algorithm is proposed in this paper. First, all gray levels are used as thresholds, so the histogram of the original image is divided into 256 small regions, and each region corresponds to one gray level. Then, two adjacent regions are merged in each iteration by a new designed scheme, and a threshold is removed each time. To improve the accuracy of the merger operation, variance and probability are used as energy. No matter how many the thresholds are, the time complexity of the algorithm is stable at *O*(*L*). Finally, the experiment is conducted on many MR brain images to verify the performance of the proposed algorithm. Experiment results show that our method can reduce the running time effectively and obtain segmentation results with high accuracy.

## 1. Introduction

Image segmentation plays an important role in computer vision and image analysis. In deep image processing, segmentation methods with high quality and efficiency can meet the requirements of the time and quality of the whole operation. The threshold segmentation algorithm has been widely studied because of its simple and effective characteristics. In most of the threshold segmentation methods, images are segmented by mining the information which is carried by the histogram. The classical threshold segmentation algorithms, such as the Otsu method [1], the minimum error method [2], and the maximum entropy method [3], have been widely used by researchers because of their high accuracy.

When single-threshold segmentation cannot meet the further operation, we need to extend the single-threshold algorithm to multithreshold algorithm. However, the time complexity will be increased exponentially with the increase of the thresholds, which limits its application in real time and cannot be tolerated. Many researchers have proposed lots of improvements [4–6], but these still cannot change the exhaustive method. Although each possibility can be obtained by the exhaustive method, it is a waste of time. In order to reduce the time complexity of the multithreshold algorithm, we proposed a novel segmentation algorithm based on histogram region merging (Algorithm 1).

In our method, each gray level is regarded as a threshold, and one threshold is removed in each iteration until the number of thresholds meets the requirement. This way, when a single threshold is obtained, the algorithm would get its maximum number of iteration times which is only 255. According to the method presented in this paper, the time complexity will be stabilized at *O*(*L*). This will be a great innovation. The details of the algorithm are introduced in the rest of the paper, and experiments are conducted to verify the performance of the proposed algorithm.

## 2. Histogram Region Merging Algorithm

The core idea of histogram region merging is to achieve the goal by reducing the number of thresholds gradually. Firstly, all gray levels in the histogram are regarded as thresholds, so the histogram is divided into 256 parts. Then, one threshold is removed in each iteration. This process can be considered as picking out a region and merging it with one of its adjacent regions each time. Through the continuous merge operation, small regions will be merged into a large region. Finally, the segmentation results are obtained from the whole histogram.

In the merging operation, more attention must be paid to the accuracy of the region selection. In order to improve the accuracy of the segmentation result, this paper will analyze the condition of selection and merger.

**Figure 1:**
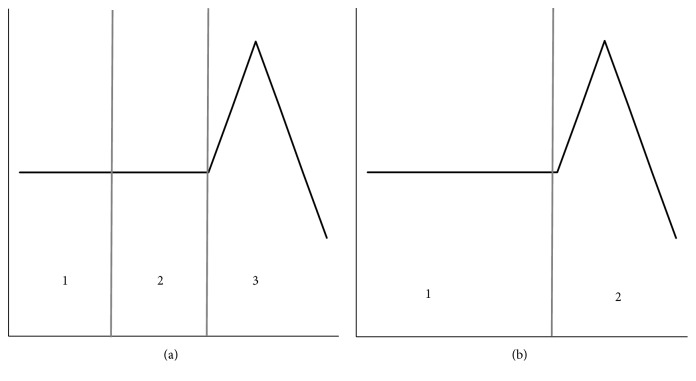
Variance.

**Figure 2:**
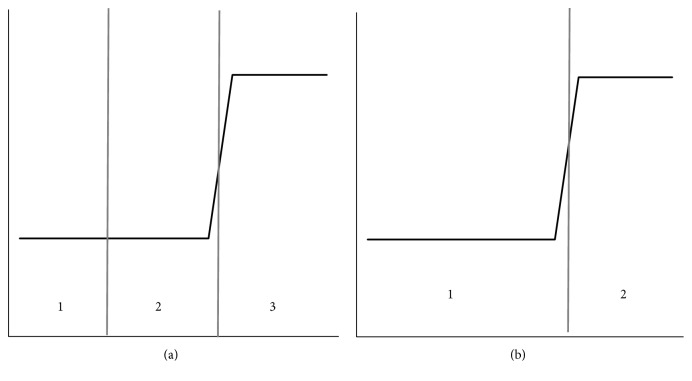
Probability.

The variance in Otsu method will be used as a reference in this paper. Variance is a measure of the uniformity of the histogram gray distribution [7]. The smaller the variance is, the better the uniformity of the gray distribution is. The visual effect shown in the histogram will change slowly. This indicates that the information carried by this part changes gently, and the part is not important in the whole segmentation. So, a priority merger should be assigned to this region.  Figure 1 displays an example of variance. In Figure 1(a), regions 1 and 2 are given priority to merge. To improve the accuracy of segmentation, both the degree of change and the size of amount are taken into account for the information. Regions with less information should be merged with other regions to form a new region which carries more information. So the probability representing the amount of information is introduced [8].  Figure 2 shows an example of the probability. In Figure 2(a), regions 1 and 2 are given priority to merge. Considering the influence of information on segmentation, the product *D* of change and amount is used as the search condition.

The formulas are as follows:

(1) ωk=∑i=tk+1tk+1Piμk=∑i=tk+1tk+1iPiωkσk2=∑i=tk+1tk+1i−μk2PiωkDk=ωkσk2,

where *P*_*i*_ is the probability of gray *i*, *P*_*i*_ = *i*/(*M* × *N*). *ω*_*k*_ is the probability of each region. *μ*_*k*_ is the gray mean value of each region. *t*_*k*_ is the threshold. *σ*_*k*_^2^ is the variance of each region. *k* is the number of regions, *k* = 0,1, 2,….

In a region, if its *D* was small, this indicates that the information is less and the change is smooth. This state proves that this region is not suitable to be a class alone in segmentation. Therefore, the region with the minimum *D* will continue to perform the merge operation.

## 3. Algorithm Process

In the proposed algorithm, the histogram is initialized to 256 small regions of size 1. Then, the region with the smallest *D* will be selected in each iteration, and it is merged with one of its adjacent regions whose *D* is smaller. So, a threshold is removed each time. Until the number of thresholds *T* meets the requirement, the iteration is stopped.

The smallest *D* is selected in each iteration. This indicates that this region is not suitable to be a part of the results, and it should be merged with other regions. After constant iterations, the smooth area will form a whole, and the sharp part will be further segmented. Each region of results will carry enough information.

## 4. Experimental Results and Analysis

In this paper, all experiments are performed on a PC with an Intel Core i3-4150, 3.5 GHz CPU, 4 GB RAM, VS2008 and opencv2.3.1.

*Variance* and* probability* are used as the energy in the proposed algorithm, while* Otsu method* and* its improved algorithms* (including Fast Otsu [9] and PSO Otsu [10]) are used in the comparative experiment. Because the results obtained by Otsu and improved Otsu are the same, we only list the segmentation images by Otsu.

100 MR images are used in this experiment. The number of thresholds is varying from 1 to 5. In this paper, 4 images and their corresponding segmentation results are given, and the number of thresholds is 3. So, the advantages of the algorithm are shown by experimental data directly in the following analysis.

**Figure 3:**
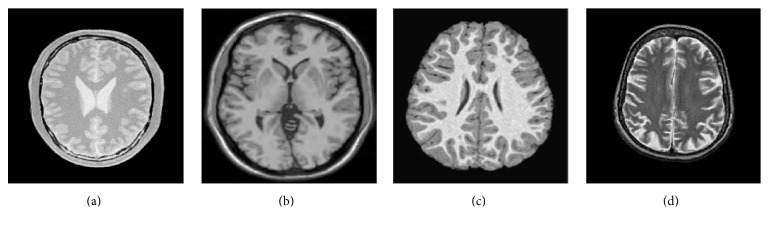
Original images.

**Figure 4:**
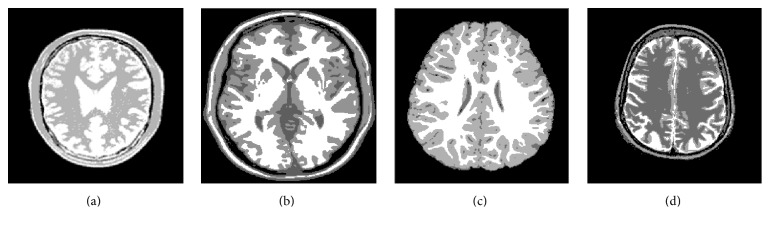
Segmentation results obtained by Otsu.

**Figure 5:**
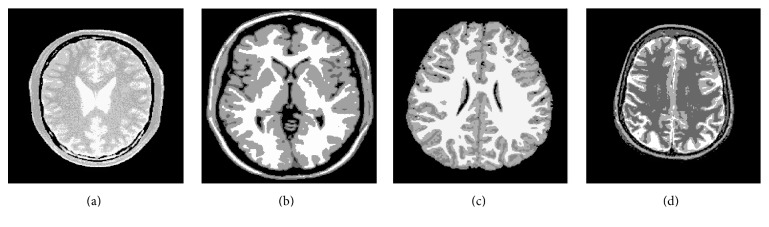
Segmentation results obtained by histogram region merging.

**Figure 6:**
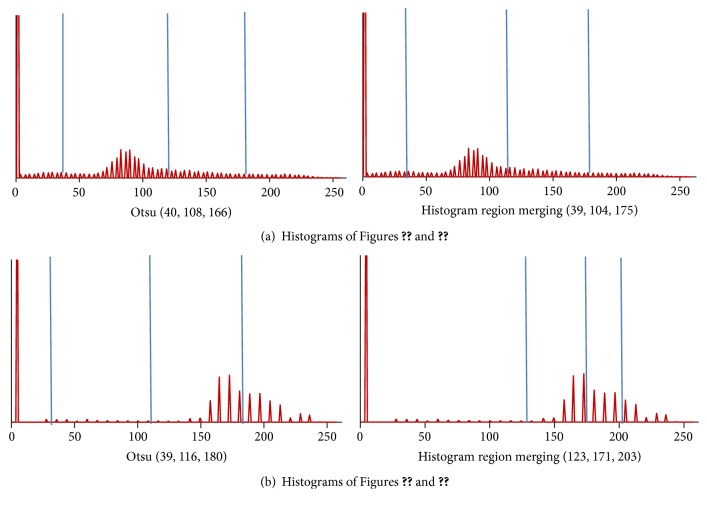
Thresholds shown in histogram.

Figure 3 displays 4 original images. Figures 4 and 5 give the segmentation results obtained by Otsu and the histogram region merging based multithreshold segmentation algorithm, respectively. It can be visually observed that the segmented images by this paper are better than classic Otsu method. The effect in image (a) is evident particularly.  Figure 6 shows the accuracy of the algorithm by the histograms of Figures 3(a) and 3(d). In Figure 6(a), the thresholds obtained by Otsu are (40, 108, 166), and the thresholds obtained by this paper are (39, 104, 175). The thresholds with high accuracy have been obtained by two methods. In Figure 6(b), thresholds (39, 116, 180) obtained by Otsu are wrong. These thresholds show that the region between thresholds 36 and 116 generates error. But thresholds (123, 171, 203) got by this paper generate better segmentation. The target region is further segmented. So, this method can avoid some error segmentations in traditional multithreshold segmentation algorithm. Thus, this method improves the accuracy of MR images segmentation.

In order to verify the effectiveness of this algorithm in data, the evaluation index *U* is used [6]. The value of *U* is closer to 1, which indicates better uniformity in the segmented image. Hence,

(2) $$\begin{array}{c} U=1-2*\left(\;P-1\;\right)*\frac{\sum_{j=1}^{P}\sum_{j\in R_{j}}^{}{\left(f_{i}-\mu_{j}\right)}^{2}}{N*{\left(f_{max}-f_{min}\right)}^{2}}, \end{array}$$

where *P* is the number of thresholds, *R*_*j*_ is the *j*th segmented region, *N* is the total number of pixels in the given image, *f*_*i*_ is gray level of pixel *i*, *μ*_*j*_ is mean gray level of pixels in *j*th region, *f*_max_ is maximum gray level of pixels in the given image, and *f*_min_ is minimum gray level of pixels in the given image.

In Table 1, it is shown that each image in this paper has a better *U* than Otsu. And the average *U* of 100 MR images proves that the accuracy of histogram region merging is not less than Otsu's. When error segmentation occurs in Otsu, this method will get an accurate result.

In Table 2, it is shown that traditional methods are time-consuming when the multithreshold segmentation is carried out. By changing the calculation or using intelligent optimization algorithm, these two ways cannot solve this problem. In this paper, no matter how many the thresholds are, the runtime will be stable at a tiny value. And the average cost is 0.03 s, which is 500 times faster than Otsu. Histogram region merging solves the problem that the exhaustive method used by the traditional algorithm wastes time. It meets the requirement of real time.

In one-threshold segmentation, Otsu just calculates 255 times. It is efficient and reaches its minimum running time. And no matter how many thresholds there are, the running time of this paper is nearly the same as that of one-threshold Otsu.

In this section, both segmented images and experimental data are better than Otsu and its improved methods. This experiment proves that the algorithm in this paper is better than the traditional multithresholds algorithm.

## 5. Conclusion

Image segmentation is important in image processing. However, the traditional multithreshold segmentation is very time-consuming. To overcome this shortcoming, we design a new scheme, named histogram region merging, which removes the thresholds one by one. Variance and probability are used to represent the change and amount of information. This not only ensures the high accuracy of region merging, but also makes the time complexity stable at *O*(*L*). No matter how much the thresholds increase, the runtime of the algorithm is tiny, and this method can avoid the phenomenon of error segmentation in Otsu. The algorithm proposed in this paper has achieved a breakthrough in the reduction of time complexity. Although the algorithm has achieved good experimental results, it still needs to continue to improve.

## Figures and Tables

**Algorithm 1:**
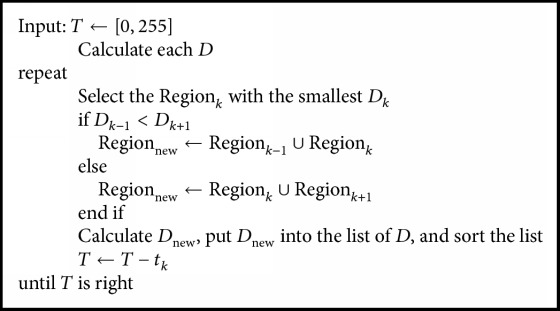
Histogram region merging.

**Table 1:** Evaluation.

Images	Otsu	This paper
a	0.9656	0.9866
b	0.9722	0.9768
c	0.9841	0.9856
d	0.9803	0.9809

Average of 100 images	0.9786	0.9804

**Table 2:** Runtime.

Images	Otsu	Fast Otsu	PSO Otsu	This paper
a	17.674 s	3.625 s	0.965 s	0.018 s
b	14.264 s	2.996 s	1.967 s	0.022 s
c	17.093 s	3.965 s	2.823 s	0.038 s
d	16.318 s	4.112 s	1.842 s	0.047 s
